# Effectiveness of a Home-Based Rehabilitation Program After Total Hip Arthroplasty Driven by a Tablet App and Remote Coaching: Nonrandomized Controlled Trial Combining a Single-Arm Intervention Cohort With Historical Controls

**DOI:** 10.2196/14139

**Published:** 2020-04-27

**Authors:** Annet Wijnen, Jildou Hoogland, Tjerk Munsterman, Carina LE Gerritsma, Baukje Dijkstra, Wierd P Zijlstra, Johan S Dekker, Janneke Annegarn, Francisco Ibarra, Geranda EC Slager, Wiebren Zijlstra, Martin Stevens

**Affiliations:** 1 Department of Orthopedics University of Groningen University Medical Center Groningen Groningen Netherlands; 2 Department of Physiotherapy Martini Hospital Groningen Groningen Netherlands; 3 Department of Orthopedics Martini Hospital Groningen Groningen Netherlands; 4 Department of Orthopedics Medical Center Leeuwarden Leeuwarden Netherlands; 5 Department of Orthopedics Ommelander Ziekenhuis Groep Scheemda Netherlands; 6 Collaborative Care Solutions Philips Research Eindhoven Netherlands; 7 University of Trento Trento Italy; 8 Department of Physical Therapy School of Health Care Studies Hanze University of Applied Sciences Groningen Netherlands; 9 Institute of Movement and Sport Gerontology German Sport University Cologne Cologne Germany

**Keywords:** remote coaching, internet, osteoarthritis, total hip arthroplasty, home-based rehabilitation program, physiotherapy, usual care, tablet app, total hip replacement, rehabilitation

## Abstract

**Background:**

Recent technological developments such as wearable sensors and tablets with a mobile internet connection hold promise for providing electronic health home-based programs with remote coaching for patients following total hip arthroplasty. It can be hypothesized that such a home-based rehabilitation program can offer an effective alternative to usual care.

**Objective:**

The aim of this study was to determine the effectiveness of a home-based rehabilitation program driven by a tablet app and remote coaching for patients following total hip arthroplasty.

**Methods:**

Existing data of two studies were combined, in which patients of a single-arm intervention study were matched with historical controls of an observational study. Patients aged 18-65 years who had undergone total hip arthroplasty as a treatment for primary or secondary osteoarthritis were included. The intervention consisted of a 12-week home-based rehabilitation program with video instructions on a tablet and remote coaching (intervention group). Patients were asked to do strengthening and walking exercises at least 5 days a week. Data of the intervention group were compared with those of patients who received usual care (control group). Effectiveness was measured at four moments (preoperatively, and 4 weeks, 12 weeks, and 6 months postoperatively) by means of functional tests (Timed Up & Go test and the Five Times Sit-to Stand Test) and self-reported questionnaires (Hip disability and Osteoarthritis Outcome Score [HOOS] and Short Form 36 [SF-36]). Each patient of the intervention group was matched with two patients of the control group. Patient characteristics were summarized with descriptive statistics. The 1:2 matching situation was analyzed with a conditional logistic regression. Effect sizes were calculated by Cohen *d*.

**Results:**

Overall, 15 patients of the intervention group were included in this study, and 15 and 12 subjects from the control group were matched to the intervention group, respectively. The intervention group performed functional tests significantly faster at 12 weeks and 6 months postoperatively. The intervention group also scored significantly higher on the subscales “function in sport and recreational activities” and “hip-related quality of life” of HOOS, and on the subscale “physical role limitations” of SF-36 at 12 weeks and 6 months postoperatively. Large effect sizes were found on functional tests at 12 weeks and at 6 months (Cohen *d*=0.5-1.2), endorsed by effect sizes on the self-reported outcomes.

**Conclusions:**

Our results clearly demonstrate larger effects in the intervention group compared to the historical controls. These results imply that a home-based rehabilitation program delivered by means of internet technology after total hip arthroplasty can be more effective than usual care.

**Trial Registration:**

ClinicalTrials.gov NCT03846063; https://clinicaltrials.gov/ct2/show/NCT03846063 and German Registry of Clinical Trials DRKS00011345; https://tinyurl.com/yd32gmdo

## Introduction

With an ageing population and increasing numbers of people with overweight and obesity, the incidence of hip osteoarthritis in the Western world will continue to rise. A further increase in the number of total hip arthroplasties is consequently expected. At present, total hip arthroplasty (THA) is considered one of the most successful clinically and cost-effective surgical treatments available for end-stage osteoarthritis, and a total of 29,937 primary THAs were performed in the Netherlands in 2017 [1]. As in other Western countries, there is an increasing tendency in the Netherlands to perform fast-track surgery, which allows people to leave the hospital within a few days. The downside is a risk of patients being minimally supported in their rehabilitation process during hospital admission and after discharge. At present, postoperative physiotherapy is not covered by Dutch basic health insurance [2]. Patients who want postoperative physiotherapy need additional insurance or have to pay for it themselves, which can lead to suboptimal recovery [3]. To optimize recovery, Bandholm and Kehlet [3] highlighted the need for immediate and intensive postoperative physiotherapy. Austin et al [4] showed that this physiotherapy does not need to take place in a formal setting, and that a home-based program could also be safe and efficacious for a majority of patients undergoing THAs. Additionally, a systematic review by Coulter et al [5] found that physical exercises for patients after THA are similarly effective whether they are performed unsupervised at home or in an outpatient setting.

Recent technological developments such as wearable sensors and tablets with mobile internet access hold promise for providing home-based programs [6]. These developments also allow for more remote coaching options. Remote coaching appears to be a good home-based rehabilitation alternative to supervised physiotherapy in an outpatient setting [6]. A home-based rehabilitation program delivered by means of videos on a tablet could therefore be helpful in the further development of such programs for patients after THA. In a previous study, we proved that such programs are feasible for patients following THA [7]. The results showed good adherence to the program and a positive patient experience; however, the effectiveness has not yet been investigated. Therefore, the aim of this study was to determine the effectiveness of this home-based rehabilitation program by comparing it with usual care. It was hypothesized that a home-based rehabilitation program could be an effective alternative to usual care.

## Methods

### Study Design

Existing data of two studies were combined in which patients of a single-arm intervention study were matched with historical controls of an observational study. First, a prospective cohort study was conducted applying a home-based rehabilitation program following THA in the Netherlands (tablet study). The study was approved by the medical ethics committee of University Medical Center Groningen (METc2014/399). Next, a transnational prospective observational trial was conducted to compare the effectiveness of the rehabilitation approach following THA in Germany versus the Netherlands (observational study). For this analysis, we used data of the Dutch patients. A protocol of this study has been published and was approved by the medical ethics committee of University Medical Center Groningen (METc2015/483) [8].

### Study Population

The tablet study (intervention group) included a total of 30 patients aged 18-75 years who received THA as treatment for primary or secondary osteoarthritis. Patients were waiting for a THA at either Martini Hospital Groningen or Medical Center Leeuwarden in the Netherlands. Exclusion criteria were: (1) revision surgery, (2) medical conditions that disallow independent living, (3) cognitive impairment, and (4) low proficiency in reading and understanding Dutch. Patients were included between December 2015 and February 2017.

The observational study (control group) included a total of 33 Dutch patients aged 18-65 years who received THA as treatment for primary or secondary osteoarthritis. Patients were waiting for a THA at either Ommelander Hospital Winschoten/Delfzijl or Medical Center Leeuwarden in the Netherlands. Exclusion criteria were: (1) medical conditions that disallow safe participation in a rehabilitation program, (2) cognitive impairment, and (3) insufficient proficiency in reading and understanding Dutch. Patients were included between March 2016 and December 2017.

### Intervention

#### Tablet Study

The home-based rehabilitation program started in the first postoperative week. The program lasted for 12 weeks and has been described in detail elsewhere [7]. Patients performed the exercises independently at home using the tablet for instructions, which were provided by means of a Web-based app [9]. The app also gave participants feedback on their training performance. The program included strengthening and walking exercises based on increasing muscle force, balance, and functionality. Exercises comprised movements that trained the abductors, flexors, and extensors of the affected hip. The content of the program was based on previous research [10,11] and on the most recent guidelines from the American Association of Orthopedic Surgeons and the Royal Dutch Society for Physical Therapy [12] (see Multimedia Appendix 1 for detailed content of the rehabilitation program).

Remote coaching was provided via weekly telephone support from a physiotherapist. During these phone calls, the physiotherapist and patient evaluated the progress and agreed on whether to train at a higher level. The program consisted of 12 levels with the aim of increasing the difficulty level each week. During the intervention, the physiotherapist made three home visits. On the first visit, participants received an explanation about the exercises and use of the tablet. The second and third visits were conducted on weeks 4 and 12 postoperatively, and included physical tests and filling out questionnaires.

#### Observational Study

The Dutch patients in the observational study received only normal usual care with no specific intervention. Both the Dutch Orthopedic Association and the Royal Dutch Society for Physical Therapy recommend continuing physiotherapeutic exercise in an extramural setting after hospital discharge to improve physical functioning [13,14]. However, as reimbursement of treatment costs depends on the insurance situation of the patient, the amount of postoperative physiotherapy applied varies among patients.

### Outcome Measures

The measurements in both the tablet study and the observational study were the same. Preoperative demographic data, height, and weight, and perioperative and postoperative complications were recorded. Both objective and self-reported measurements were taken to assess mobility, functional status, and quality of life of the patients. Measurements were taken preoperatively and postoperatively at 4 weeks, 12 weeks, and 6 months at the patients’ homes.

#### Objective Measurement

To assess mobility and functional status objectively, the Timed Up & Go (TUG) test and the Five Times Sit-to Stand Test (FTSST) were performed. TUG is an accepted test to measure mobility, and is considered reliable and practical [15]. During the TUG test, participants were instructed to stand up from the chair, walk 3 meters, turn around, walk back, and sit down on the chair again. Participants were asked to walk at a fast but safe pace. The test was performed three times.

The FTSST is a clinical test to assess lower extremity power and balance, which shows good reliability and validity [16,17]. For the FTSST, participants were asked to stand up and sit down 5 times at a fast speed. The participants were instructed to perform the test with their arms crossed in front of the abdomen when possible.

#### Self-Reported Measurements

The self-reported Hip disability and Osteoarthritis Outcome Score (HOOS) was used as a disease-specific outcome measure of functional status and quality of life. The HOOS consists of five subscales: pain, other symptoms, function in activities of daily living, function in sport and recreational activities, and hip-related quality of life. Standardized response options are given and each question is scored from 0 to 4 on a 5-point Likert scale. A normalized score ranging from 0 to 100 is subsequently calculated for each subscale (with 0 indicating extreme symptoms and 100 indicating no symptoms). The Dutch version of the HOOS has proven to be valid and reliable [18].

To measure health-related quality of life, the Short Form 36 (SF-36) was used, which is a widely used generic health status questionnaire consisting of 36 questions divided into eight health concepts: physical functioning, role limitations due to physical problems, social functioning, bodily pain, general mental health, role limitations due to emotional problems, vitality, and general health perceptions. Each raw scale score is transformed into a linear 0-100 scale, in which higher scores indicate less disability. In this study, only the subscales physical functioning, role limitations due to physical problems, and general health perceptions were analyzed. The SF-36 has proven to be practical, reliable, and valid for a general and chronic disease population [19].

### Data Analysis

Data were analyzed using IBM SPSS Statistics version 23 (IBM Corp, Armonk, NY, USA) and Statistical Analysis System v. 9.4 (SAS Institute, Cary, NC, USA). Patient demographics were analyzed with descriptive statistics using mean and SD or frequency and percentage as appropriate.

This study was designed as a nonrandomized controlled trial combining existing data of two studies. This was achieved by matching each patient in the intervention group to two patients in the control group (ie, 1:2 matching) [20]. Patients in the intervention group were aged 18-75 years, whereas none of the patients in the control group was older than 65 years. Therefore, intervention group patients older than 65 years were excluded from the analysis. The remaining 15 patients in the intervention group were matched based on gender and age to two patients in the control group. Since there were three matches of a woman with a man, these matches were excluded from the analyses. The 1:2 matching situation was analyzed with conditional logistic regression, which is an extension of logistic regression that takes matching into account. A correction for baseline was performed as there were significant differences at baseline between the three groups (intervention group and two matching control groups). Significance was tested at alpha=.05.

Effect sizes were calculated between the intervention group and control groups using Cohen *d*. As one intervention group patient was matched with two control group patients, two effect sizes were calculated per intervention group patient. Cohen *d* of 0-0.19, 0.20-0.49, 0.50-0.79, 0.80-1.29, and >1.3 represents no or a negligible effect, small effect, medium effect, large effect, and very large effect, respectively [21].

## Results

### Demographic Characteristics

In total, 15 patients of the intervention group and 15 and 12 patients of each control group were included in the analyses. The baseline characteristics of the three groups are presented in Table 1. No significant differences between the groups were found. The intervention group completed approximately 2-9 times more hours of exercise during the 12 weeks of the home-based rehabilitation program compared to that completed by the control groups in the 6 months after surgery.

Baseline characteristics and functional scores on all outcomes of the three groups are presented in Table 1 and Table 2, respectively. Regarding the functional measurements, the intervention group performed the FTSST significantly faster at baseline than the two control groups. For three of the five subscales of the HOOS, the intervention group performed significantly better at baseline than the control groups. Similarly, the intervention group showed higher scores on two subdomains of the SF-36: physical functioning and general health perception (Table 2).

**Table 1:** Baseline characteristics of the study population (N=42).

Characteristic		Intervention group (n=15)	Control group 1 (n=15)	Control group 2 (n=12)	*P* value
Gender: female, n (%)		10 (67)	10 (67)	7 (58)	.85^a^
Age (years), mean (SD)		59.3 (3.6)	59.3 (3.6)	59.3 (5.3)	>.99^b^
Body mass index (kg/m^2^), mean (SD)		26.7 (5.1)	28.0 (4.3)	31.1 (6.5)	.10^b^
**Educational level, n (%)**					.08^b^
	Low	3 (20)	7 (46)	4 (33)	
	Medium	6 (40)	5 (33)	8 (67)	
	High	6 (40)	3 (20)	0 (0)	
**Living situation, n (%)**					.58^a^
	Alone	4 (27)	2 (13)	1 (8)	
	With partner and/or children	11 (73)	13 (87)	11 (92)	
**ASA** ^c^ **classification, n (%)**					.12^a^
	I or II	13 (87)	12 (80)	6 (50)	
	III	2 (13)	3 (20)	6 (50)	
Exercise hours^d^, median (range)		17.9 (13.1-19.9)	6.0 (0.0-48.0)	2.5 (0.0-48.0)	N/A^e^

^a^Fisher exact test.

^b^One-way analysis of variance.

^c^ASA: American Society of Anesthesiologists Physical Status Classification System.

^d^Based on 12 weeks of the program for the intervention group and 6 months postoperative for the control groups.

^e^N/A: not applicable.

**Table 2:** Functional outcome measures at baseline of the study population (N=42).

Measurement		Intervention group (n=15)		Control group 1 (n=15)		Control group 2 (n=12)		Odds ratio (95% CI)^a^		*P* value	
TUG^b^ (seconds), mean (SD)		11.3 (2.6)		12.0 (4.6)		13.2 (5.3)		0.92 (0.77-1.10)		.33	
FTSST^c^ (seconds), mean (SD)		16.2 (3.0)		20.8 (6.6)		23.8 (11.0)		0.83 (0.70-1.00)		.04	
**HOOS^d^, mean (SD)**											
	Pain	48.9 (12.8)		35.5 (14.8)		36.3 (18.4)		1.12 (1.03-1.23)		.03	
	Other symptoms	50.0 (18.2)		29.3 (13.1)		41.7 (19.5)		1.07 (1.01-1.13)		.03	
	Function in ADL^e^	52.7 (17.5)		34.0 (10.2)		37.1 (19.1)		1.14 (1.03-1.27)		.02	
	Function in sport and recreational activities	23.3 (13.7)		16.3 (10.0)		20.8 (22.1)		1.04 (0.98-1.11)		.16	
	Hip-related quality of life	19.2 (10.7)		22.9 (12.9)		24.5 (13.5)		0.98 (0.93-1.03)		.43	
**SF-36^f^, mean (SD)**											
	Physical functioning	42.7 (14.4)		28.0 (15.2)		24.6 (19.1)		1.07 (1.01-1.13)		.02	
	Role limitations: physical	33.3 (40.8)		20.0 (36.8)		14.6 (24.9)		1.01 (0.99-1.03)		.16	
	General health perception	80.9 (12.8)		67.9 (17.4)		51.6 (25.5)		1.08 (1.01-1.16)		.03	

^a^Conditional logistic regression.

^b^TUG: Timed Up & Go test.

^c^FTSST: Five Times Sit-to Stand Test.

^d^HOOS: Hip disability and Osteoarthritis Outcome Score; scale of 0-100 (0 = extreme symptoms, 100 = no symptoms).

^e^ADL: activities in daily living.

^f^SF-36: 36-item Short Form Health Survey; scale of 0-100 (higher score = better perceived health or functioning).

### Objective Measurements

The outcomes of the objective measurements are shown in Table 3. After baseline correction, significant differences were found in the TUG at 4 weeks, 12 weeks, and 6 months, and on the FTSST at respectively 12 weeks and 6 months. The intervention group performed the two measurements significantly faster than the two control groups.

### Self-Reported Measurements

The outcomes of the self-reported measurements are presented in Table 4. The scores of the intervention group were slightly higher on both disease-specific outcome measures of functional status and health-related quality of life, but the differences were not significant. For the HOOS, the intervention group scored significantly better on the subdomain function in sport and recreational activities at 4 weeks, 12 weeks, and 6 months, and also scored significantly higher on the subdomain hip-related quality of life after 6 months. For the SF-36, the intervention group scored significantly better on the subdomain physical role limitations at 12 weeks and 6 months.

### Effect Sizes

Effect sizes of the outcome measures at week 12 and at the 6-month follow up are presented in Table 5. The home-based rehabilitation program had a medium to very large effect on the FTSST and TUG test at 12 weeks (range Cohen *d*=0.6-1.5) compared to usual care. These effects were still present at the 6-month follow-up measurement (range Cohen *d*=0.5-1.2). Regarding the self-reported outcomes, the effect sizes at 12 weeks ranged from small to very large. In particular, function in activities of daily living for the HOOS and physical functioning for the SF-36 showed large and very large effect sizes. At the 6-month follow up, a large or very large effect was found on all subdomains of the HOOS and SF-36, except for general health perception of the SF-36.

**Table 3:** Objective outcome measures of the study population (N=42).

Measurement		Intervention group (n=15)	Control group 1 (n=15)	Control group 2 (n=12)	Odds ratio (95% CI)^a^	*P* value
**TUG^b^ (seconds), mean (SD)**						
	T1^c^	10.5 (2.1)	14.8 (4.0)	13.3 (3.9)	0.68 (0.48-0.97)	.04
	T2^d^	8.0 (1.0)	10.3 (2.6)	10.0 (3.4)	0.34 (0.14-0.84)	.02
	T3^e^	7.5 (1.0)	9.02 (1.9)	8.9 (2.5)	0.33 (0.13-0.86)	.02
**FTSST^f^ (seconds), mean (SD)**						
	T1	14.9 (2.9)	21.3 (4.5)	17.7 (3.5)	0.75 (0.56-1.00)	.05
	T2	12.6 (1.9)	16.7 (2.4)	14.9 (3.1)	0.49 (0.24-0.99)	.05
	T3	11.7 (1.5)	14.7 (2.5)	14.0 (2.4)	0.56 (0.31-0.99)	.05

^a^Results of conditional logistic regression, corrected for baseline.

^b^TUG: Timed Up & Go test.

^c^T1: 4 weeks.

^d^T2: 12 weeks.

^e^T3: 6 months.

^f^FTSST: Five Times Sit-to-Stand Test.

**Table 4:** Mean (SD) of self-reported outcome measures of the study population (N=42).

Measure			Intervention group (n=15)	Control group 1 (n=15)	Control group 2 (n=12)	Odds ratio (95% CI)^a^	*P* value
**HOOS^b^**							
	**Pain**						
		T1^c^	88.8 (7.6)	71.7 (17.1)	73.5 (15.9)	1.24 (0.93-1.64)	.14
		T2^d^	94.0 (3.4)	87.9 (10.7)	78.5 (16.6)	1.19 (0.98-1.45)	.09
		T3^e^	98.7 (2.1)	85.1 (12.5)	85.6 (11.3)	1.29 (0.94-1.77)	.12
	**Other symptoms**						
		T1	75.3 (13.2)	68.0 (18.9)	62.1 (14.1)	1.03 (0.97-1.09)	.33
		T2	82.3 (10.0)	78.7 (15.8)	74.6 (13.1)	1.04 (0.96-1.13)	.37
		T3	91.0 (6.0)	76.3 (13.0)	77.5 (17.7)	1.10 (1.00-1.21)	.06
	**Function in ADL^f^**						
		T1	76.5 (8.0)	60.6 (17.0)	58.1 (15.3)	1.22 (0.94-1.57)	.13
		T2	92.8 (8.5)	79.0 (11.3)	69.7 (14.8)	1.24 (0.99-1.55)	.06
		T3	96.8 (3.6)	80.0 (15.6)	79.1 (15.6)	1.25 (0.94-1.67)	.13
	**Function in sport and recreational activities**						
		T1	70.0 (18.8)	26.7 (20.1)	29.7 (24.3)	1.10 (1.01-1.20)	.04
		T2	76.3 (20.5)	63.8 (26.1)	49.1 (21.6)	1.04 (1.00-1.07)	.04
		T3	82.5 (11.6)	59.6 (19.3)	64.9 (24.1)	1.09 (1.01-1.17)	.03
	**Hip-related quality of life**						
		T1	50.8 (15.1)	45.8 (18.3)	43.2 (19.5)	1.04 (0.99-1.10)	.11
		T2	75.4 (14.8)	71.3 (19.7)	63.0 (21.4)	1.03 (0.99-1.07)	.21
		T3	88.8 (8.9)	71.3 (20.2)	69.3 (22.5)	1.09 (1.02-1.17)	.02
**SF-36^g^**							
	**Physical functioning**						
		T1	54.8 (15.9)	41.1 (20.2)	36.3 (20.6)	1.04 (1.00-1.08)	.09
		T2	86.0 (6.8)	62.6 (19.2)	538 (18.0)	1.30 (0.89-1.91)	.17
		T3	89.0 (7.1)	65.0 (18.9)	59.2 (20.8)	1.26 (0.99-1.60)	.07
	**Role limitations: physical**						
		T1	20.0 (34.3)	23.3 (29.1)	6.3 (15.5)	1.01 (0.99-1.04)	.35
		T2	70.0 (42.5)	38.3 (42.1)	37.5 (34.5)	1.04 (1.00-1.08)	.04
		T3	93.3 (25.8)	56.7 (44.8)	45.8 (39.7)	1.04 (1.00-1.08)	.03
	**General health perception**						
		T1	83.1 (15.3)	77.4 (15.9)	66.9 (17.3)	1.00 (0.94-1.07)	.91
		T2	86.1 (13.5)	73.9 (20.8)	63.0 (22.2)	1.05 (0.97-1.13)	.26
		T3	84.8 (21.5)	72.6 (14.8)	63.4 (31.8)	1.01 (0.97-1.07)	.57

^a^Conditional logistic regression adjusted for baseline for comparison of the two control groups and intervention group.

^b^HOOS: Hip disability and Osteoarthritis Outcome Score; scale of 0-100 (0 = extreme symptoms, 100 = no symptoms).

^c^TI: 4 weeks.

^d^T2: 12 weeks.

^e^T3: 6 months.

^f^ADL: activities in daily living.

^g^SF-36: 36-item Short Form Health Survey; scale of 0-100 (higher score = better perceived health or functioning).

**Table 5:** Cohen *d* (95% CI) based on mean (SD) functional measure values corrected for baseline.

Measurement		Twelve weeks		Six months	
		Intervention group vs control group 1	Intervention group vs control group 2	Intervention group vs control group 1	Intervention group vs control group 2
TUG^a^		**1.2**^b^ (0.4-1.9)	0.7 (–0.1-1.4)	**1.1** (0.3-1.8)	0.6 (–0.2-1.4)
FTSST^c^		**1.5** (0.6-2.2)	0.6 (–0.2-1.3)	**1.2** (0.4-2.0)	0.5 (–0.3-1.3)
**HOOS^d^**					
	Pain	0.6 (–0.2-1.3)	**1.4** (0.5-2.2)	**1.4** (0.6-2.2)	**1.4** (0.5-2.2)
	Other symptoms	0.0 (–0.7-0.7)	0.5 (–0.3-1.2)	**0.9** (0.1-1.6)	**0.9** (0.1-1.7)
	Function in ADL^e^	**1.2** (0.4-1.9)	**1.9** (1.0-2.8)	**1.3** (0.4-2.0)	**1.3** (0.5-2.1)
	Function in sport and recreational activities	0.5 (0.2-1.3)	**1.2** (0.3-1.9)	**1.2** (0.4-1.9)	**0.9** (0.1-1.7)
	Hip-related quality of life	0.2 (–0.5-0.9)	0.6 (–0.2-1.4)	**1.0** (0.2-1.7)	**1.2** (0.3-1.9)
**SF-36**					
	Physical functioning	**1.3** (0.5-2.1)	**1.8** (0.8-2.6)	**1.2** (0.4-1.9)	**1.5** (0.6-2.2)
	Role limitations: physical	**0.8** (0.1-1.6)	**0.9** (0.0-1.6)	**0.9** (0.1-1.6)	**1.2** (0.3-1.9)
	General health perception	0.2 (–0.5-0.9)	0.2 (–0.6-0.9)	0.1 (–0.6-0.8)	–0.1 (–0.8-0.7)

^a^TUG: Timed Up & Go test.

^b^Effect sizes of *d*>0.80 are in bold.

^c^FTSST: Five Times Sit-to-Stand Test.

^d^HOOS: Hip disability and Osteoarthritis Outcome Score.

^e^ADL: activities in daily living.

^f^SF-36: 36-item Short Form Health Survey.

## Discussion

The aim of this study was to determine the effectiveness of a home-based rehabilitation program delivered by means of internet technology. To that end, the effectiveness of the program was compared with usual care in the Netherlands. It was hypothesized that a home-based rehabilitation program could be an effective alternative to usual care.

Significant differences were found considering the objective outcomes, and the home-based rehabilitation program also seemed to have had large to very large effects on the TUG test and FTSST at the end of the 12-week program. These large effect sizes in favor of the intervention group were still present at the 6-month follow-up measurement. These results are further supported by the self-reported outcomes. In particular, function in activities of daily living for the HOOS and physical functioning for the SF-36 showed very large effect sizes. At the 6-month follow-up measurement, a large or very large effect was found on all subdomains of the questionnaires, with a medium to large effect found only on the general health perception domain of the SF-36. It can therefore be concluded that compared to usual care, the home-based rehabilitation program has a large to very large effect on disease-specific outcome measures and quality of life.

Austin et al [4] demonstrated that a rehabilitation program does not need to take place in a formal setting to be effective. Our study further shows that recent technological developments can be helpful in providing such a home-based rehabilitation program for patients after THA. Another advantage is that owing to recent technological developments, our program can start immediately after surgery, as advised by Bandholm and Kehlet [3]. Furthermore, earlier research found that physical exercises are similarly effective whether they are performed unsupervised at home or supervised in an outpatient setting [4,5]. Our results show that a home-based rehabilitation program can also be effective when offered with the help of modern technology.

Although the home-based rehabilitation program was performed unsupervised at home, the Web-based app gave participants feedback on their training performance, and remote support was provided through weekly telephone contact with a physiotherapist. A systematic review by Geraedts et al [6] showed that remote coaching in home-based rehabilitation programs is a good alternative to supervised physiotherapy in an outpatient setting. The results of our study are in line with this conclusion. During these phone calls, the physiotherapist and patient evaluated the progress and agreed on whether to train at a higher level. Hoogland et al [7] investigated the feasibility of this home-based rehabilitation program, showing that patients appreciated the weekly telephone-based remote support. The importance of this weekly telephone contact is in line with a previous study of Silveira et al [22] showing that motivation and coaching are important factors for home-based exercise performance and enhanced adherence. Hoogland et al [7] found good adherence to this home-based rehabilitation program and a positive patient experience. This is also in line with other studies indicating that telerehabilitation leads to high levels of patient satisfaction [23,24].

In demonstrating large effects in the intervention group, the results of our study imply that a home-based rehabilitation program after THA can be more effective than usual care. It can therefore be concluded that such a program is an effective alternative to formal physiotherapy. As patients in the Netherlands need additional insurance for physiotherapy or have to pay for it themselves, this could be an option to offer every patient a certain amount of physiotherapy. Although the cost-effectiveness has not yet been determined, a home-based rehabilitation program is likely to be more cost-effective than usual care. A physiotherapist will work for fewer hours, without compromising the effectiveness of the rehabilitation program for patients. In addition, a home-based rehabilitation program can be more suitable than usual care for (1) elderly people who cannot come to the physiotherapy practice by themselves; (2) people living in remote, rural areas who are not always able to travel far; and (3) people who live independently and are not allowed to travel by car the first 6 weeks after surgery.

A limitation of the study is the small number of patients, although this was deliberately chosen as it was a pilot study. However, this small number of patients did not limit finding large effect sizes. In addition, patients who had agreed to participate in the intervention were expected to be able to complete the home-based rehabilitation program, as we wanted to test the intervention for the first time. It is therefore possible that there was selection bias, as patients of the intervention group were probably more motivated than the average patient. Nonetheless, the wide variety in educational level, age, and living situation seems to have provided a representative group. Although there were differences between the intervention group and control groups, these were corrected for in our analyses. Lastly, since patients in the Netherlands need additional insurance for physiotherapy or have to pay for it themselves, there is variability in health care consumption within usual care, resulting in a heterogeneous control group in the current study; some patients received no physiotherapy at all, while others received up to 48 hours of physiotherapy. This variability is representative of the current situation in the Netherlands.

In conclusion, our results clearly demonstrate larger effects in the intervention group, implying that a home-based rehabilitation program after THA can be more effective than usual care. In future research, it would be interesting to conduct a randomized controlled trial with a larger sample size and where at least the outcome assessor is blinded. In addition, it would be worthwhile to investigate whether the home-based rehabilitation program is also effective for people older than 65 and suitable for patients with low preoperative physical functioning. Cost-effectiveness should also be assessed.

## Appendix

Multimedia Appendix 1 Content of the home-based rehabilitation program.

## Abbreviations

FTSST: Five Times Sit-to Stand Test
HOOS: Hip disability and Osteoarthritis Outcome Score
SF-36: 36-item Short Form Health Survey
THA: total hip arthroplasty
TUG: Timed Up & Go test
