# Supplementary material for: Effectiveness of a Home-Based Rehabilitation Program After Total Hip Arthroplasty Driven by a Tablet App and Remote Coaching: Nonrandomized Controlled Trial Combining a Single-Arm Intervention Cohort With Historical Controls
Source: JMIR Rehabil Assist Technol. 2020 Apr 27;7(1):e14139. doi: 10.2196/14139 (PMC7215512; doi:10.2196/14139)
Supplement: Multimedia Appendix 1 [file rehab_v7i1e14139_app1.docx]

**Multimedia Appendix 1:** Content of the home-based rehabilitation program.

|  | **Strength exercises** | | **Walking/Step exercise** | |
| --- | --- | --- | --- | --- |
|  | **(Days 1, 3 & 6)** | | **(Days 2 & 5)** | |
|  | **Exercise** | **Intensity** | **Exercise** | **Intensity** |
| **Level 1** | Sitting knee extensions (operated leg) | 2 x 10 reps | Walking | 3x 5 min |
|  | Standing knee raise (operated leg) | “ |  |  |
|  | Heel and toe raises | “ |  |  |
|  | Hip extension (operated leg) | “ |  |  |
|  | Hip abduction (operated leg) | “ |  |  |
| **Level 2** | Sitting knee extensions (operated leg) | 3 x 10 reps | Walking | 2x 10 min |
|  | Standing knee raise (operated leg) | “ |  |  |
|  | Heel and toe raises | “ |  |  |
|  | Hip extension (operated leg) | “ |  |  |
|  | Hip abduction (operated leg) | “ |  |  |
|  | Bilateral mini-squat behind a chair | 2 x 10 reps |  |  |
| **Level 3** | Heel and toe raises | 3 x 10 reps | Walking | 2x 10 min |
|  | Sitting knee extensions (operated leg) | “ |  |  |
|  | Standing knee raise (operated leg) | “ | Step | 2x 10 reps |
|  | Hip extension (operated leg) | “ |  |  |
|  | Hip abduction (operated leg) | “ |  |  |
|  | Bilateral mini-squat behind a chair | 3 x 10 reps |  |  |
| **Level 4** | Heel and toe raises | 3 x 10 reps | Walking | 3x 10 min |
|  | Sitting knee extensions (operated leg) | 2 x 10 reps + ankle weight (1/2 kg) |  |  |
|  | Standing knee raise (operated leg) | “ | Step | 2x 10 reps |
|  | Hip extension (operated leg) | “ |  |  |
|  | Hip abduction (operated leg) | “ |  |  |
|  | Bilateral mini-squat behind a chair | 3 x 10 reps |  |  |
| **Level 5** | Heel and toe raises | 3 x 10 reps | Walking | 3x 10 min |
|  | Sitting knee extensions (operated leg) | 3 x 10 reps + ankle weight (1/2 kg) |  |  |
|  | Standing knee raise (operated leg) | “ | Step | 3x 10 reps |
|  | Hip extension (operated leg) | “ |  |  |
|  | Hip abduction (operated leg) | “ |  |  |
|  | Bilateral mini-squat behind a chair | 3 x 10 reps |  |  |
| **Level 6** | Tandem stance, one hand for support | 2 x 10 sec | Walking | 2x 15 min |
|  | Heel and toe raises | 3 x 10 reps |  |  |
|  | Chair rise/sit to stand | 2 x 5 reps | Step | 3x 10 reps |
|  | Standing knee raise (operated leg) | 3 x 10 reps + ankle weight (1/2 kg) |  |  |
|  | Hip extension (operated leg) | “ |  |  |
|  | Hip abduction (operated leg) | “ |  |  |
| **Level 7** | Tandem stance, one hand for support | 2 x 15 sec | Walking | 2x 15 min |
|  | Heel and toe raises | 3 x 10 reps |  |  |
|  | Chair rise/sit to stand | 3 x 5 reps | Step | 2x 15 reps |
|  | Standing knee raise (operated leg) | 3 x 10 reps + ankle weight (1/2 kg) |  |  |
|  | Hip extension (operated leg) | “ |  |  |
|  | Hip abduction (operated leg) | “ |  |  |
|  | Hip abduction (non-operated leg) | “ |  |  |
| **Level 8** | Tandem stance, one hand for support | 2 x 15 sec | Walking | 2x 20 min |
|  | Heel and toe raises | 3 x 10 reps |  |  |
|  | Chair rise/sit to stand | 3 x 5 reps | Step | 2x 15 reps |
|  | Single-leg stance, one hand for support | 2 x 10 sec |  |  |
|  | Standing knee raise (operated leg) | 3 x 15 reps + ankle weight (1/2 kg) |  |  |
|  | Hip extension (operated leg) | “ |  |  |
|  | Hip abduction (operated leg) | “ |  |  |
|  | Hip abduction (non-operated leg) | “ |  |  |
| **Level 9** | Tandem stance, one hand for support | 2 x 20 sec | Walking | 2x 20 min |
|  | Heel and toe raises | 3 x 10 reps |  |  |
|  | Chair rise/sit to stand | 3 x 5 reps | Step | 3x 15 reps |
|  | Single-leg stance, one hand for support | 4 x 10 sec |  |  |
|  | Standing knee raise (operated leg) | 3 x 15 reps + ankle weight (1 kg) |  |  |
|  | Hip extension (operated leg) | “ |  |  |
|  | Hip abduction (operated leg) | “ |  |  |
|  | Hip abduction (non-operated leg) | “ |  |  |
| **Level 10** | Tandem stance, without hand support | 2 x 20 sec | Walking | 1x 25 min |
|  | Heel and toe raises | 3 x 10 reps |  |  |
|  | Chair rise/sit to stand | 3 x 5 reps | Step | 3x 15 reps |
|  | Single-leg stance, one hand for support | 4 x 15 sec |  |  |
|  | Standing knee raise (operated leg) | 3 x 15 reps + ankle weight (1 kg) |  |  |
|  | Hip extension (operated leg) | “ |  |  |
|  | Hip abduction (operated leg) | “ |  |  |
|  | Hip abduction (non-operated leg) | “ |  |  |
| **Level 11** | Tandem stance, without hand support | 2 x 25 sec | Walking | 1x 25 min |
|  | Heel and toe raises | 3 x 10 reps |  |  |
|  | Chair rise/sit to stand | 3 x 5 reps | Step | 4x 15 reps |
|  | Single-leg stance, without hand support | 4 x 15 sec |  |  |
|  | Standing knee raise (operated leg) | 4 x 15 reps + ankle weight (1 kg) |  |  |
|  | Hip extension (operated leg) | “ |  |  |
|  | Hip abduction (operated leg) | “ |  |  |
|  | Hip abduction (non-operated leg) | “ |  |  |
| **Level 12** | Tandem stance, without hand support | 2 x 25 sec | Walking | 1x 30 min |
|  | Heel and toe raises | 3 x 10 reps |  |  |
|  | Chair rise/sit to stand | 4 x 5 reps | Step | 4x 15 reps |
|  | Single-leg stance, without hand support | 4 x 20 sec |  |  |
|  | Standing knee raise (operated leg) | 4 x 15 reps + ankle weight (1 kg) |  |  |
|  | Hip extension (operated leg) | “ |  |  |
|  | Hip abduction (operated leg) | “ |  |  |
|  | Hip abduction (non-operated leg) | “ |  |  |
| Abbreviations: minutes (min); seconds (sec); repetitions (reps). | | | | |
